# Cutting force measurement: Hand tool instrumentation used in slaughterhouses – a systematic review

**DOI:** 10.17179/excli2021-3167

**Published:** 2021-04-13

**Authors:** Salvador Francisco Tirloni, Adriana Seára Tirloni, Nestor Roqueiro, Eugenio Andrés Díaz Merino, Giselle Schmidt Alves Díaz Merino, Antônio Renato Pereira Moro

**Affiliations:** 1 Technological Center, Federal University of Santa Catarina, SC, Brazil; 2 Automation and Systems Engineering, Federal University of Santa Catarina, SC, Brazil; 3 Communication and Expression Center, Federal University of Santa Catarina, SC, Brazil

**Keywords:** tool, knife, ergonomics, force, meat packing, slaughterhouse

## Abstract

Workers' intensive use of hand tool cutting in the meat packing industry is a risk factor for occupational health, mainly by mechanical compression of tissues in the upper limbs, which can cause Work-Related Musculoskeletal Disorders (WMSDs). This systematic review aimed to identify the characteristics and measured variables of instrumented knives and determine how they should be designed. The review process and article extractions occurred through an analysis of the (article) titles, keywords and abstracts, followed by reading the full texts by two reviewers independently. Searches were conducted in Medline, Web of Science, Science Direct, Scopus, Ebsco and Engineering Village for articles published in peer-reviewed journals from January 2000 to March 2019, in the English language. The result of (the) search included 1289 potentially eligible studies, with 894 duplicated/triplicated/quadruplicated articles that were excluded, resulting in 404 remaining articles of which 33 were considered eligible, with 36 additional articles, totaling 69 evaluated full texts. After the review, none of the 14 analyzed studies, were rated as having good methodological quality. In addition, four types of instrumented knives were used. Data acquisition was performed in both laboratory and meat processing plants. It is noteworthy that only one knife was submitted to a validation process and that the articles did not provide complete technical information about the knives. The result demonstrated that the cutting force varies within and between subjects, tasks, plants and blade finishings. All knives used some type of electrical connection via cable or wires. Of the articles found, none considered the influences that the workers are subject to when they do not use the same tool daily for data acquisition. Therefore, the development of different types of instrumented knives, with wireless data transmission and more rigorous studies are necessary to expand the knowledge of the cutting force and development of WMSD in slaughterhouse workers who perform meat cutting.

## Introduction

Statistically, Brazil is the world's largest exporter of chicken (ABPA, 2020[2]) and beef (ABIEC, 2020[1]), and pork occupies fourth place (ABPA, 2017[2]). Consequently, Brazil employs many workers in this sector, according to OSHA (2013[25]), where several occupational risk factors are present: repetitive work, artificially cold environments, use of gloves and hand tools, resulting in the application of force in the tasks.

As stated by the Brazilian Work Accident Statistics report, in the period between 2015 and 2017, the economic sector of slaughtering pigs, poultry, and other small animals was 3^rd^ place among the sectors that most developed occupational diseases in workers at the national level, with the cattle sector in 9^th^ place (Ministério da Fazenda, 2017[19]).

## Work-Related Musculoskeletal Disorders (WMSDs)

Ergonomics-related risk factors that may lead to the development of WMSDs in poultry processing facilities include, among other factors, the amount of physical effort to perform a demanding task (such as heavy lifting, hanging/rehanging poultry, pulling skin) or to maintain control of equipment or tools (OSHA, 2013[25]).

Workers must use a knife that is sized and designed for the task performed (OSHA, 2013[25]). Besides, the type, shape and texture of the knife grip should be appropriate for the hand of the worker and the eventual use of gloves (Ministério do Trabalho e Emprego, Brasil, 2013[20]). According to the Brazilian norm (NR-36) regarding slaughterhouses (Ministério do Trabalho e Emprego, Brasil, 2013[20]), the employer must implement a control system for sharpening knives, establishing mechanisms for the constant replenishment of sharp knives.

Several studies checked that poultry slaughterhouse workers were exposed to moderate risk, which meant an incidence of upper-limb work-related musculoskeletal disorders (UL-WMSDs) from 10.6 to 21.5 % (Reis et al., 2016[30], 2017[32], 2019[31]). The Brazilian standard for slaughterhouses refers to the fact that the process organization and the speed of the production line must take into account the time variability required by different productions and processes, at least the time required for knife sharpening operations (Ministério do Trabalho e Emprego, Brasil, 2013[20]).

The force demand is considered a risk factor to developing UL-WMSDs on the OCRA Checklist method and this assessment is carried out using the Borg Scale (Colombini and Occhipinti, 2014[5]). In one study, most slaughterhouse workers perceived tool sharpening as very sharp (63.1), however, the exertion applied when cutting the leg, breast and sassami (cleaning) as mild (48.7 %) and moderate (42.1 %). In general, 54 % of the workers felt discomfort in their upper limbs, 38.8 % in the shoulder and 28.9 % in the hand (Tirloni et al., 2019[36]). Szabo et al. (2001[35]) state that too little reconditioning has the undesirable consequence of increasing forceful exertions and effort needed to accomplish the manual cutting task. In their study, when reconditioning took place after every 6 cutting cycles for the high-force job and 9 cutting cycles for low-force, knife dulling increased cutting force by 15 % for the same cut in carrageenan gel as compared with a fresh knife. The cutting force increased by 30 % after 13 and 21 cycles of the high- and low-force jobs, respectively.

As there was a considerable variation among workers in sharpness differences, Dempsey and McGorry (2004[6]) suggest that one potential administrative control would be to maintain knife sharpness so that exposure is minimized, and another option would be more frequent substitution of knives for freshly-sharpened ones. 

In Brazil, the Ministry of Agriculture, Livestock and Food Supply - Brazil recommends the sterilization of knives and scissors at least twice a shift. Nevertheless, as found by Tirloni et al. (2019[36]), the tools were sharpened and sterilized four times throughout the workday in a specific room. Karltun et al. (2016[8]) verified that the median value of knife usage time for the 12 individuals varied from about 1 h 20 min to almost 3 h. For a working day of 8 h, the meat cutters in this study needed 3-6 freshly sharpened knives/ day to be able to perform their work and be content with the sharpness of their knives during the entire day. In the study of Dempsey and McGorry (2004[6]), there was variation among workers in sharpness differences between the initial reading and 2- and 5-hour readings.

Due to studies that confirm the effect of knife sharpening on the cutting force, it is suggested to follow the recommendations of OSHA (2013[25]), where employers train poultry slaughterhouse employees on tool sharpening, maintenance schedules, and good cutting techniques, to assure that knives, scissors, and other tools used for cutting are sharp and workers do not exert excessive force. Along these lines, Marsot et al. (2007[11]) declared that suitable training of knife users should be carried out to achieve best possible cutting performance. Claudon and Marsot (2006[4]) highlight the importance of training operators in knife honing/sharpening to ensure they have knives that cut easily. In addition, when analyzing the cut in a carrageenan gel, Szabo et al. (2001[35]) revealed that significant force increases may be anticipated for too infrequent reconditioning, in which this increase may increase fatigue onset and the risk of WMSDs.

Despite the guidelines, several studies cited that knife use is a risk for health workers (OSHA, 2013[25]; Ministério do Trabalho e Emprego, Brasil, 2013[20]), the slaughterhouse workers had a greater chance of feeling cold in the hands (Tirloni et al., 2018[37]). Increased cutting force may intensify fatigue onset and the risk of WMSD (Szabo et al., 2001[35]; Dempsey and McGorry, 2004[6]). The use of a badly sharpened knife can increase upper limb biomechanical stresses (Claudon and Marsot, 2006[4]). Although most poultry slaughterhouse workers perceived the tool's sharpness as very sharp (63.1 %), most determined that the exertion applied when cutting the product as mild (48.7 %), as well as moderate (42.1 %) (Tirloni et al., 2019[36]).

Moreover, the meat cutters had an extremely high prevalence of disorders in wrists/hands and shoulders (Arvidsson et al., 2012[3]). Tirloni et al. (2018[37]) analyzed some studies on the discomfort of slaughterhouse workers and verified that hand discomfort was cited by 18-29 % of them.

Finally, one study applied the Occupational Repetitive Action (OCRA) method, with 101 participants from three slaughterhouses. The workers were asked to evaluate the perceived effort (Borg scale) if they are cutting the meat with a badly sharpened knife and a very sharp one. It was possible to identify the influence of the knife edge on the risk of developing musculoskeletal disorders and it was found that there was a significant increase (29 %) of this risk when the knife is "badly sharpened". Therefore, maintaining well-sharpened knives for optimal performance of the cutting task (fewer technical actions) is suggested, as well as including knife sharpening in the standard operating procedure (Tirloni et al., 2020[38]).

## Study Questions

For McGorry (2001[12]), the lack of field data is due, in part, to the current inadequacy of instrumentation measurement and its apparent lack is widespread. Additionally, this absence of data makes it difficult to study the potentially causal relationship between risk factors and injury, to validate the redesign of tasks, or to identify high-risk techniques for performing a specific task.

Therefore, many reasons justify this systematic review, because it detects the technologies used in the instrumented knives and the limitations of the studies; making it possible to create guidelines to build an instrumented knife that can reliably measure the force applied during cutting. In addition, there is a gap regarding quantitative data on the cutting force to cut animals of different sizes in slaughterhouses, considering the complexity of the cut and the state of meat/piece (freezing level and meat with or without bone).

This systematic review aimed to identify the characteristics and measured variables of instrumented knives, and to determine how they should be designed.

This study had the following specific research questions:

a) What are the characteristics of instrumented hand cutting tools?

- was the tool used the same as or different from those used in the company surveyed?
- was data transmission wired or wireless?
- has the tool weight changed?
- what was the data acquisition environment?

b) What variables can be obtained by these instruments? 

c) How should instrumented hand cutting tools be designed?

## Materials and Methods

### Search strategy

Searches were conducted in six electronic databases in international journals in the areas of Engineering and Health Sciences: Pubmed (Medline), Web of Science, Science Direct, Scopus, EBSCO (Medline complete), and Compendex (Engineering Village); in the English language with publications from January 2000 to March 2019. Additional records were identified in the screening step of this research through an analysis of the articles' references included for eligibility. 

The principles of PICO were used (population, intervention, comparison, and outcomes) to group the search terms. As this study was not clinical research, three principles were considered and two Boolean operators were used (OR, AND): population (“meat processing industry” OR “meat processing plant” OR “meat industry” OR “meat packing” OR “meat cutting” OR slaughterhouse OR abattoir) AND intervention (cutting OR tool OR hand OR handle OR knife OR sharp) AND outcomes (force OR effort OR exertion OR strength OR “upper limb” OR “musculoskeletal disorders” OR ergonomics). 

The search terms were defined based on the list of terms used in MeSH Database (NCBI, 2019[23]). The studies were collected in databases of 28-29 March 2019 and explored the word roots and found all term variants (singular/plural, past tense, gerund, comparative and superlative adjective; when possible). The following filters were used for the subject area: ergonomics, medicine, engineering (industrial, biomedical, electrical electronic, manufacturing and mechanical), robotics, health professions, material science, multidisciplinary and public environmental occupational health, according to availability in the database.

#### Inclusion and exclusion criteria

The eligible studies contained the following criteria: (1) performed study with cutting manual tool; (2) the tool should be an instrumented knife; (3) written in English; (4) full text papers published in peer-reviewed journals; and (5) with adult humans (+19 years old).

The exclusion criteria were studies that used apparatus with blade or knife, not a hand tool; those that approached cutting meat using robots; and finally, no studies with cutting manual task using knife. The search results were exported to EndNote^®^ basic software online, where duplicates/triplicates/quadruplicates were removed, data extraction was obtained in full text after the analysis of the possible eligibility of the articles.

#### Study analysis

In relation to the studies' eligibility, the review process occurred through an analysis of the titles, keywords and reading the abstracts by two reviewers independently in EndNote^®^ basic software online. When in doubt of eligibility, the full text was reviewed. In the cases of any disagreement between the two reviewers, a decision was reached by consensus, or a third researcher provided further review and the decision was made by arbitration.

#### Methodological quality assessment of studies

The quality of the eligible studies was assessed by tools proposed by the United States National Institutes of Health (NIH) (NIH, 2018[24]). This study included the tools to evaluate the Quality Assessment of Controlled Intervention Studies and Cross-Sectional Studies (both with 14 criteria). The NIH website (NIH, 2018[24]) provides the assessment tools and guidance for determining the quality of each type of study containing explanatory information about each item that should be analyzed in the paper.

The quality rating was classified as good, fair, or poor, allowing the general analysis of the evaluators considering all the items (NIH, 2018[24]). Each item of the assessment tool chosen, depending on the type of study, received a positive appraisal (+) when the study attended the item, negative (-) when it did not attend as well as other options (cannot determine - CD, not applicable - NA and not reported - NR).

According to Wong et al. (2008[41]), observational studies with rating ≥ 67 % of the positive item attended were an indication of good quality, with 34-66 % of the positive checks were of fair quality, and ≤ 33 % of poor quality. The quality studies that allowed internal and external validity criteria were used (Sanderson et al., 2007[33]).

#### Data extraction

Two independent reviewers accomplished the data extraction and review process, in cases of disagreement; consensus was reached through discussion between them or through arbitration with a third reviewer. 

The following study's characteristics were extracted and described: authors' names, title of the article, publication year; country where the study was conducted; design and objective of the study; characteristics of participants; environment of data acquisition (laboratory or company); what product was cut; type of hand cutting knife (same as used by workers in the company, wireless or not, weight); outcomes measured by the knife; follow up and results. Data of this study were presented descriptively and followed the PRISMA Statement for reporting flow diagram (Moher et al., 2009[21]), and the NIH checklist for systematic reviews (NIH, 2018[24]).

## Results

**Figure 1:**
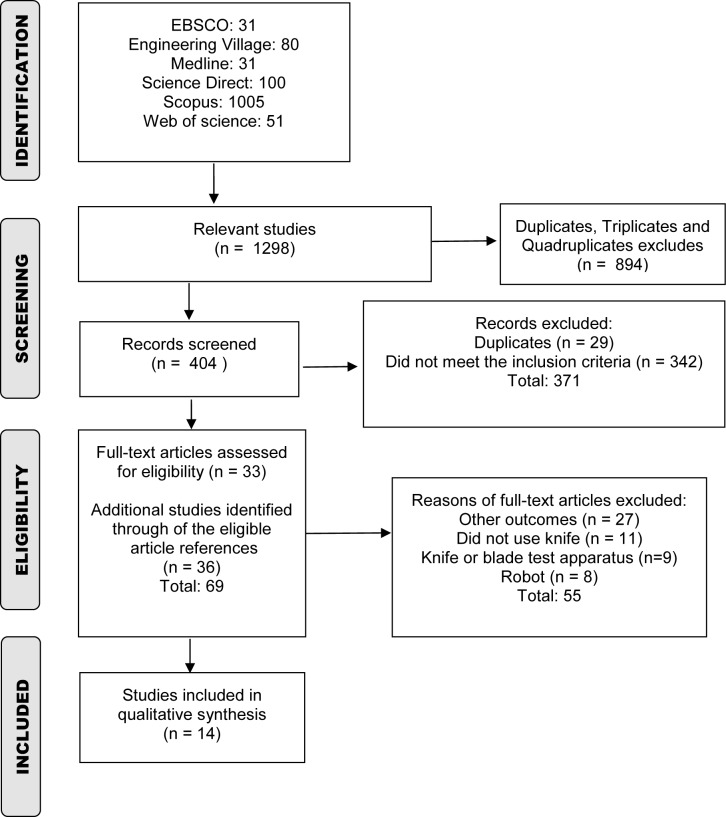
Flow diagram of systematic review process

The search results included 1298 potentially eligible studies. Firstly, 894 duplicated/triplicated/quadruplicated articles were excluded and, of the 404 remaining articles, 33 were considered eligible based on the review of the titles, keywords, and abstracts. Additional studies (36) were included after searching in the eligible article references, totaling 69 full texts for evaluation. After reviewing them, 55 studies were ineligible, ending the process with 14 studies for quality assessment (Figure 1(Fig. 1)).

The collections of the 14 final studies were performed only in a laboratory (6), in meat packing and meat processing plants (6) and in both environments (2), involved four types of instrumented knives, additionally, included three countries: United States, New Zealand and Denmark (Table 1(Tab. 1); References in Table 1: Dempsey and McGorry, 2004[6]; Juul-Kristensen et al. 2002[7]; McGorry, 2001[12]; McGorry et al., 2000[18], 2003[16], 2004[14][13], 2005[17][15]; Murphy et al., 2000[22]; Pontonnier et al., 2011[27], 2012[26], 2014[28]; Waddell et al., 2003[39]). Only two studies' design types were found in this review, however, the majority were controlled intervention studies (9) (as randomized, a randomized trial, a randomized clinical trial, or a Randomized Control Trial - RCT) (Table 1(Tab. 1)). Four types of knives were identified by the study, which will be detailed later in Table 3.

Table 2(Tab. 2) (References in Table 2: Dempsey and McGorry, 2004[6]; Juul-Kristensen et al., 2002[7]; McGorry, 2001[12]; McGorry et al., 2000[18], 2003[16], 2004[14][13], 2005[17][15]; Murphy et al., 2000[22]; Pontonnier et al. 2011[27], 2012[26], 2014[28]; Waddell et al., 2003[39]) presents the assessment of the methodological quality of studies. The analysis showed that most of the papers have fair (11) methodological quality and do not have research with good classification. In the control intervention studies, it is negatively highlighted that all studies mentioned randomizing the variables (subjects/groups/ tools/height/knives/force level/dynamometer/trials/blades), but did not cite how it was done; the evaluations and treatment were not blinded, the studies did not calculate the sample size and test power. Thus, in the cross-sectional studies, the population was not specified, the participation rate of eligible persons was less than half; the sample size justification was not provided; the variable measures were not obtained prior to the outcomes; the time frame was insufficient to see an association between exposure and outcome; a single assessment was made; the outcome assessors were not blinded, and finally, potentially confounding variables were not measured and adjusted statistically.

Four instrumented knives were found in the review, where IK-A was the most used in the research (9) and the only ones that had gone through a validation process, are described in Murphy et al. (2000[22]). All knives used some type of electrical connection via cable or wire and were not completely the same as the tool used daily by the worker. During data collection, if there was a module attached to the knife user's body, no study cited battery life (hours, days) or any kind of radio frequency transmission. The tool characteristics are described in Table 3(Tab. 3) (References in Table 3: Juul-Kristensen et al., 2002[7]; Madeleine et al., 1999[10]; McGorry, 2001[12]; McGorry et al., 2000[18], 2005[17][15]; Murphy et al., 2000[22]; Pontonnier et al., 2011[27], 2014[28]; Waddell et al., 2003[39]).

Table 4(Tab. 4) (References in Table 4: Dempsey and McGorry, 2004[6]; Juul-Kristensen et al., 2002[7]; McGorry, 2001[12]; McGorry et al., 2000[18], 2003[16], 2004[14][13], 2005[17][15]; Murphy et al., 2000[22]; Pontonnier et al., 2011[27], 2012[26], 2014[28]; Waddell et al., 2003[39]) shows the main results of the studies analyzed and the referred instrumentalized knives - IK (Instrumented Knife).

### Grip force

The grip force (standard deviation) varied among the studies found, the peak was 71.2 (20.8) N (McGorry et al., 2004[14]) and 130.9 (26.5) N (Dempsey and McGorry, 2004[6]), in addition, the mean grip force was 52.7 (15.7) N (McGorry et al., 2004[14]), 55.2 (16.2) N (McGorry et al., 2004[13]), and 39.9 (4.4) N (Dempsey and McGorry, 2004[6]). The same occurred with the peak cutting moment, it was 16.2 (3.1) Nm (Dempsey and McGorry, 2004[6]), 8.94 (2.07) Nm (McGorry et al., 2004[14]) and the mean cutting moment was 4.0 (0.6) Nm (Dempsey and McGorry, 2004[6]) and 6.30 (1.38) Nm (McGorry et al., 2004[14]). In addition, the average grip force during the task in a pork slaughterhouse were between 11 and 35 % of the maximum voluntary grip force (Dempsey and McGorry, 2004[6]). 

For McGorry (2001[12]), the working range exceeded 700 N for gripping forces, and 28 and 16 Nm for the two applied moment axes, with the upper limits of these variables being larger than the studies presented. The knife forces may vary between-subject differences by site, performing the same meat cutting tasks, within-subject differences by cut, and cut-by-site (Waddell et al., 2003[39]). Corroborating results of mean and peak cutting moments were 4.7 and 17.2 Nm for the shoulder boning, 3.5 and 12.9 Nm for the rib trim, and 2.3 and 10.6 Nm for the loin trim, respectively (McGorry et al., 2003[16]).

The study by Juul-Kristensen et al. (2002[7]) also found that there was a difference between different production processes to carry out the same task (cut chicken). They established that the cutting forces were significantly higher during the cutting task compared to the isolated cutting task. However, there was a small intra-individual coefficient of variation during the 50 cuts in the cutting task.

McGorry (2001[12]) found that the grip force and applied moment during the initial phase (25 %) of the clay cut were greater for a high-precision task than for a low-precision task, regardless of the thickness level of the clay cut. On the other hand, McGorry et al. (2000[18]) verified that the thickness of the material being cut had a significant effect on the average peak torque.

Different factors interfere in applied force; one of them is the work pace. McGorry et al. (2004[14]) certified that the production pace requiring a grip force of 58.9 (14.6) N and cutting moment of 6.79 (1.40) Nm as compared to 46.6 (14.4) N and to 5.80 (1.20) Nm for the self-pace task, respectively. Another factor is the workers' experience level of the task. For McGorry et al. (2000[18]), experienced workers spent less time applying torque than less experienced workers, but there were no between-group differences in the number of cutting cycles per trial (30 s).

### Cutting force 

Pontonnier et al. (2014[28]) verified that the range of the cutting force was 18-67 N. In the study by Juul-Kristensen et al. (2002[7]), the estimated cutting forces during manual deboning were of 6.25 N and 20.71 N (median and peak levels). 

Task and person-related factors were found to influence the power required to perform the task (McGorry et al., 2000[18]), the same differences were observed between plants for the same cut (Waddell et al., 2003[39]). In the development of cutting measurement equipment, according to McGorry et al. (2005[15]), the cutting forces for fiber mesh and the meat were very similar, however, the force required to cut the mesh increased with the number of passes through the dulling sandpaper.

McGorry et al. (2004[14]) analyzed the cutting force applied in different combinations of workstation configuration and blade angle. The results showed that with a bent blade, the required grip force was significantly higher, 54.9 (16.1) N, than when a straight blade was used, 50.5 (15.0) N. Likewise, a significant difference was also found for the effect of cut complexity, with a complex cut as 49.8 (14) N vs. a simple cut operation with 55.5 (16.8) N.

### Posture 

The the workstation configurations and the direction that the cut is made affects the working posture. One study identified that extreme positions of the hand in ulnar deviation were larger in cutting than mechanical deboning (p> 0.05) (Juul-Kristensen et al., 2002[7]).

In relation to the cut direction performed in the shoulder abduction, there was a demand between 7-30 % of joint capabilities (Pontonnier et al., 2014[28]). The height decrease of the workbench tended to lower the muscle activation levels, except for the deltoideus medialis (Pontonnier et al., 2012[26]). 

Analyzing the posture during the cutting task at four workbench heights, Pontonnier et al. (2014[28]) found that the range of motion's standard deviations were large, underlining the notably different motions from one subject to another, especially for shoulder rotation and flexion. Waddell et al. (2003[39]) proved that in one cutting task, the forearm flexor and extensor muscle activity were significantly smaller when compared to another task, but the total range of motion in this task was larger. 

The optimal bench height for the meat-cutting task requiring force of approx. 50 N, which should be between 20 and 30 cm lower than the worker's elbow height (Pontonnier et al., 2014[28]). The surface height had a significant effect on the grip force requirements, but the magnitude of the difference was around 2 N (McGorry et al., 2004[14]).

### Knife sharpness 

As reported by McGorry et al. (2005[15]), sharpness affects force exposure, but also cutting efficiency, in relation to cutting time. Blade sharpness was found to effect grip forces, cutting moments, and cutting time, with sharper blades requiring statistically and significantly lower peak and mean cutting moments, and grip forces than dull knives (McGorry et al., 2003[16]). Therefore, efforts to provide and maintain sharp blades can have a significant impact on force exposure (McGorry et al., 2003[16]). In one of the two analyzed tasks, McGorry et al. (2005[17]) found that the quality of the blade finish decreased the cutting time, the average and peak grip strength and cutting moment, however, this result was not generalized. This corroborates the findings of Waddell et al. (2003[39]) where the results of knife strength varied between the tasks analyzed under the same conditions. Dempsey and McGorry (2004[6]) verified considerable variations among workers in the sharpness differences between the initial reading and 2- and 5-hour readings, which could lead to different levels of exposure.

Based on the results of this study, the specific research questions are answered as follows:

a) Characteristics of instrumented manual cutting tools:

All knives used some type of electrical connection via cable or wires, even the most recent study (Pontonnier et al., 2014[28]).

No knife researched was completely the same as the tool used daily by workers.

All knives used for data acquisition had changes in their weight (mass). 

Of the 14 studies, 6 were exclusively in the laboratory and 6 exclusively on the factory floor, with 2 studies in both locations.

b) Variables that can be obtained by the researched knives:

Cutting force; cutting torque; cutting time; grip force and number of cutting cycles per time.

c) How should instrumented hand-cut tools be designed?

By analyzing related articles it is possible to understand which instrumentalization (action of instrumenting) characteristics can interfere during data acquisition in the real environment of developing the workers' tasks. In this sense, the instrumentalization of manual cutting tools should consider some basic points: the visual non-mischaracterization of the tool, that is, maintaining the original features of the knife. When boarding an electronic system in a tool, there should be no increase in weight or change in the center of mass. Essential questions for this approach are the use of wireless data transmission and battery charging via magnetic induction.

In this research field, there would be instrumentation entirely associated with the work tool and imperceptible by the worker. In this regard, deeper studies without adjacent variables should be performed. Reliable data acquisition of the task would be one example since the worker does not experience physical and psychological interference due to the tool's instrumentalization. Physical, in terms of obstructing the natural movement. By altering the shape of the cable and adding an electric cable or electrical connection wires would change the weight and center of mass. Psychological, in the sense of removing the spontaneity of the movement by knowing that it is being observed and measured. According to Prates and Barbosa (2003[29]), in data collection techniques, one of the challenges for evaluators is to be able to observe without interfering in the context or inhibiting the user.

## Discussion

Key indicators of the measuring instrument quality are the reliability and validity of the measurements (Kimberlin and Winterstein, 2008[9]), nevertheless, the results of this study introduced that only one knife was submitted to both processes (IK-A). The validity is the extent to which an instrument measures what it purports to measure but requires reliability (correctly calibrated) (Kimberlin and Winterstein, 2008[9]). It is noteworthy that two studies mentioned performing knife calibration but did not refer to how they did it.

The results showed that IK-A was the most used in papers (n=9), but in a short period 2000-2005, besides the experimental studies were classified as fair. Evidencing that more robust studies are needed for data generalization with better descriptions of the used tools. 

Of the fourteen studies found, half of them were developed in the laboratory. According to Scott and Renz (2006[34]), it is unlikely that scenarios developed in the laboratory will become directly applicable solutions to solve industrial problems. In this sense, there is a need for more field research (factory floor) to elucidate all the reflexes caused to the slaughterhouse worker, who uses the knife as a work tool daily.

Although more field studies are welcome to describe the worker's daily scenario more accurately, some precautions are important during data collection. Westgaard and Winkel (1997[40]) argue that the main disadvantage of conducting research in the area is that experimental research "in loco" is less controlled than in the laboratory, due to numerous exogenous factors that are beyond the researcher's control. Therefore, the use of a tool identical to that used daily by the worker can contribute to a reduced number of exogenous variables during data acquisition.

Regarding the variables obtained in the researched studies, it is observed that the new technologies were not present, for example, the measurement of movement by means of accelerometers and gyroscopes embedded in different types of cables. This form of measurement would obtain more complete answers, as it could be integrated into the production line. Thus, it would then identify what was registered in the study by Tirloni et al. (2020[38]) as a frequent additional risk factor in work completely determined by machines in 94 % of tasks.

## Limitations

One of the limitations of this study was how the articles were selected, as it was only an analysis of the data in the title, keywords and abstract that were initially reviewed. The other was that most studies carried out many experiments/analyses in the same manuscript, which did not make the methods and results description judicious. Specifically, the instrumented knife characteristics were not found, along with some information that had been identified elsewhere in the paper or other articles (knife description). Finally, the studies data were not collected over days or weeks, so conclusions are limited to the studied conditions.

## Conclusion

Four knives with instrumented handles were found in this systematic review, data acquisition was performed in laboratory and meat processing plant. It is noteworthy that only one knife was submitted to the validation process and that the articles provided incomplete technical information about the knives. The methodological quality of the studies was poor and fair. 

Although the number of instrumented knives, as well as studies on instrumented knives, was limited, some of them attempted to provide data on the effects of sharpening, strength, cutting moment and grip.

Although a classification was made to try to obtain the details of each instrumentation presented in the articles, none of them considered the influences that the workers are subject to when they do not use the same tool daily. 

The use of new technologies could promote the development of a low-cost wireless data transmission knife, where the instrumentation was imperceptible by the user, due to the absence of physical changes, maintaining the characteristics of the original knife used by the worker.

Therefore, an instrumented knife or handle should be developed to meet the requirements of the present study, as well as conducting future studies with this instrumented knife in slaughterhouses. These studies have shown the need to deepen knowledge about cutting force and the relationship with the risks of developing WMSD, plus the use of gloves, the ambient temperature of the workplace, the experience of worker, the effects of boning training, the frequency of using the knife sharpener to keep the knife sharp, and the effects on increasing technical actions.

## Conflict of interest

The authors declare that they have no conflict of interest.

## Figures and Tables

**Table 1:**
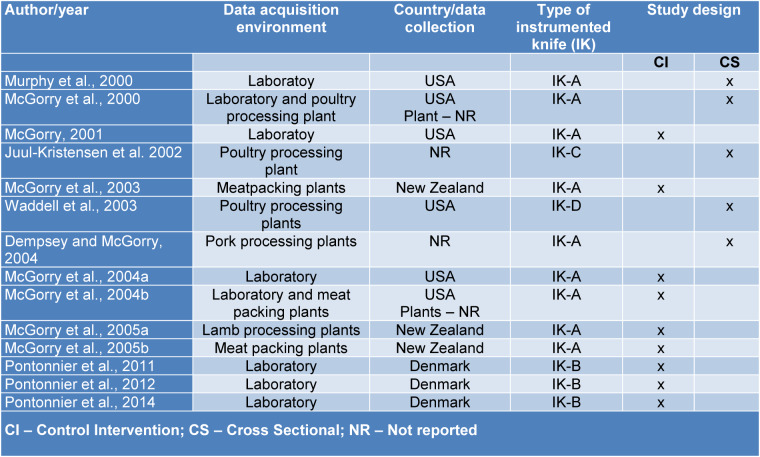
Characteristics of the study design included in this review

**Table 2:**
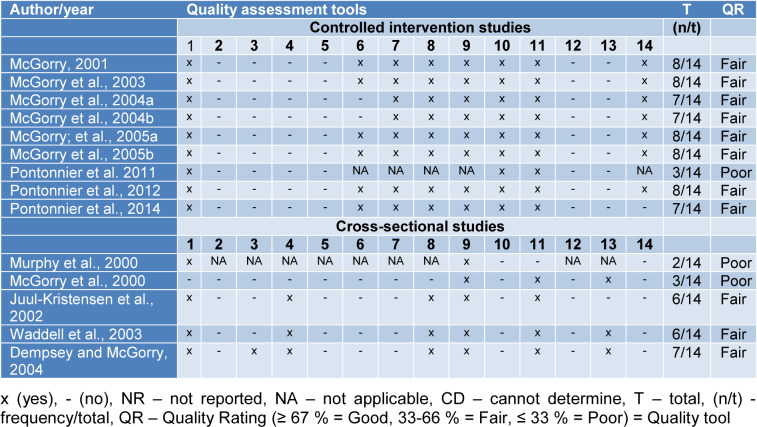
Assessment of the methodological quality of the studies

**Table 3:**
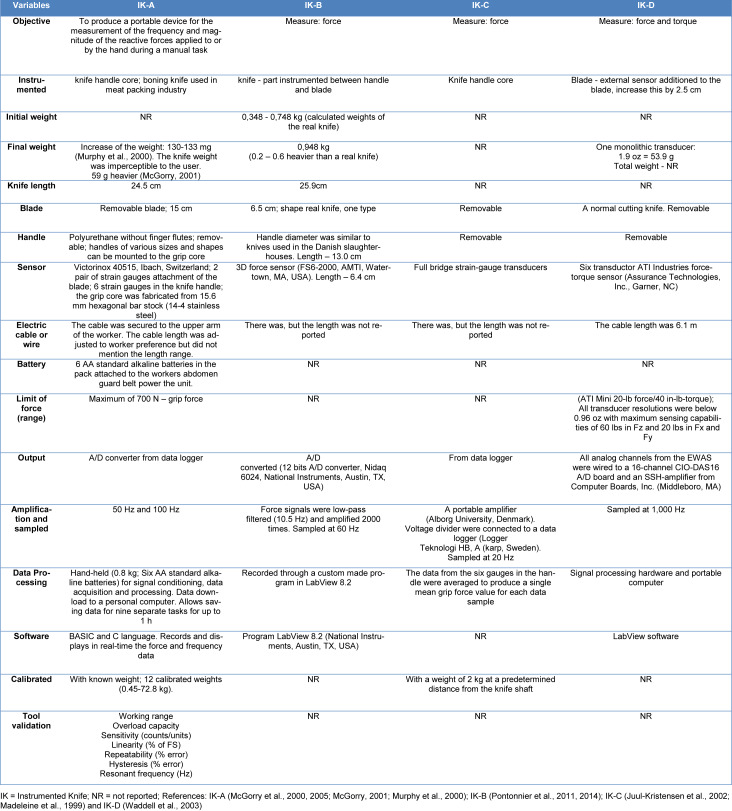
Characteristics of the instrumented knives used in the articles included in this systematic review

**Table 4:**
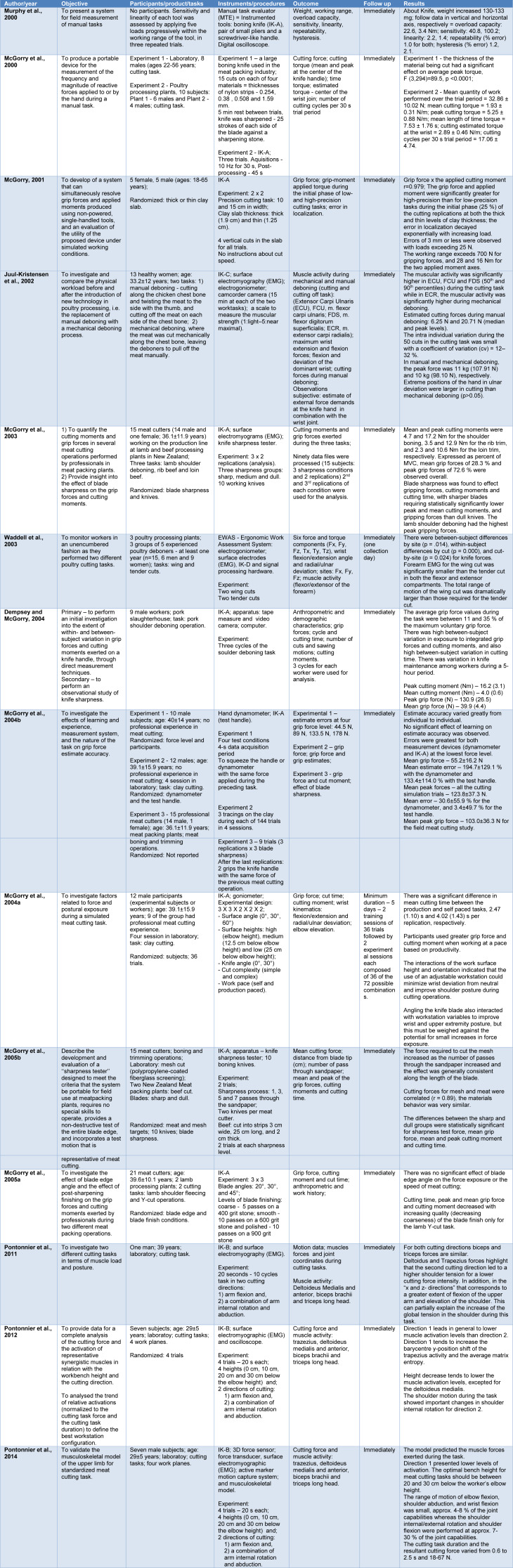
Description of the studies included in this review
